# Development of a screening eye clinic for Ebola virus disease survivors: Lessons learned and rapid implementation at ELWA Hospital in Monrovia, Liberia 2015

**DOI:** 10.1371/journal.pntd.0007209

**Published:** 2019-03-07

**Authors:** Jessica G. Shantha, Brent R. Hayek, Ian Crozier, Catherine Gargu, Robert Dolo, Jerry Brown, John Fankhauser, Steven Yeh

**Affiliations:** 1 Uveitis and Vasculitis Service, Emory Eye Center, Emory University School of Medicine, Atlanta, Georgia, United States of America; 2 Ministry of Health and Sanitation, Monrovia, Liberia; 3 ELWA Hospital, Monrovia, Liberia; 4 Emory Global Health Institute, Emory University, Atlanta, Georgia, United States of America; University of Geneva Hospitals, SWITZERLAND

## Abstract

**Background:**

In the wake of the West African Ebola virus disease (EVD) outbreak of 2014–2016, thousands of EVD survivors began to manifest a constellation of systemic and ophthalmic sequelae. Besides systemic arthralgias, myalgias, and abdominal pain, patients were developing uveitis, a spectrum of inflammatory eye disease leading to eye pain, redness, and vision loss. To investigate this emerging eye disease, resources and equipment were needed to promptly evaluate this sight-threatening condition, particularly given our identification of Ebola virus in the ocular fluid of an EVD survivor during disease convalescence.

**Methodology/Principal findings:**

A collaborative effort involving ophthalmologists, infectious disease specialists, eye care nurses, and physician leadership at Eternal Love Winning Africa (ELWA) Hospital in Liberia led to the development of a unique screening eye clinic for EVD survivors to screen, treat, and refer patients for more definitive care. Medications, resources, and equipment were procured from a variety of sources including discount websites, donations, purchasing with humanitarian discounts, and limited retail to develop a screening eye clinic and rapidly perform detailed ophthalmologic exams. Findings were documented in 96 EVD survivors to inform public health officials and eye care providers of the emerging disease process. Personal protective equipment was tailored to the environment and implications of EBOV persistence within intraocular fluid.

**Conclusions/Significance:**

A screening eye clinic was feasible and effective for the rapid screening, care, and referral of EVD survivors with uveitis and retinal disease. Patients were screened promptly for an initial assessment of the disease process, which has informed other efforts within West Africa related to immediate patient care needs and our collective understanding of EVD sequelae. Further attention is needed to understand the pathogensis and treatment of ophthalmic sequelae given recent EVD outbreaks in West Africa and ongoing outbreak within Democratic Republic of Congo.

## Author summary

The West African Ebola virus disease (EVD) outbreak from 2014–2016 resulted in thousands of EVD survivors at-risk for uveitis, an inflammatory eye condition with the potential for vision impairment or blindness if not promptly diagnosed and treated. Given the equipment and resource limitations within West Africa, particularly for subspecialty eye care, a screening eye clinic was developed at ELWA Hospital in Liberia to promptly screen, treat, and refer patients with eye disease. The implementation of a screening eye clinic in collaboration with leadership at ELWA Hospital was of immediate relevance given our limited understanding of this eye disease prior to the recent EVD outbreak. Resourceful procurement of medications and equipment, as well as the development of modular systems within an eye clinic setting, facilitated patient examination, teaching, and documentation of this spectrum of ophthalmic illness. Development of a screening eye clinic and its rapid implementation were needed to improve our collective undersanding of the spectrum of uveitis as a disease sequelae of EVD, relevant for recent and future outbreaks.

## Introduction

The West African Ebola virus disease (EVD) outbreak from 2014–2016 was of historic magnitude with over 28,600 cases and 11,300 deaths predominantly within the highest transmission countries of Liberia, Sierra Leone and Guinea.[1] This has resulted in the largest cohort of EVD survivors in history with thousands of EVD survivors requiring medical care. Following survival from acute EVD, Ebola survivors are at significant risk of systemic and ocular sequelae.[2] These sequleae include arthritis, arthralgias, headache, hair loss, abdominal pain, and uveitis. Knowledge of the ophthalmic complications identified in EVD survivors prior to this most recent outbreak was limited given the size of prior outbreaks dispersed throughout central Africa.[3] As reports of eye disease emerged via communications with providers in West Africa, in addition to the repatriation of health care workers (HCWs) from West Africa to the United States following Ebola virus (EBOV) infection[4,5], the clinical imperative for rapid screening evaluation of EVD survivors and assessment of the prevalence of eye disease, clinical spectrum, and treatment algorithms became increasingly evident.

Specifically, after our team cared for a repatriated United States health care worker (HCW) with aggressive, sight-threatening panuveitis associated with heterochromia, scleritis, and refractory hypotony following recovery from life-threatening, critical illness due to EVD, there were concerns for ophthalmic sequelae with potentially vision-threatening consequences throughout West Africa.[4,6] Another United States HCW had also developed severe uveitis requiring oral corticosteroids to avert severe vision impairment.[5] In addition, the finding of replicating Ebola virus in the ocular fluid raised the emerging public health concern of possible infectivity of ocular fluid if eye care providers performed invasive procedures on EVD survivors.[4] With increasing anecdotal reports of sight-threatening eye disease emerging from West Africa and concerns about undiagnosed and untreated eye disease at the Eternal Love Winning Africa (ELWA) EVD survivor clinic, the administrative and clinical leadership of ELWA Hospital, in partnership with clinicians from Emory Eye Center, responded to the emerging reports of ocular symptoms and eye disease in EVD survivors via the development of a screening eye clinic. This eye clinic, entitled the ‘Quiet Eye West Africa Project’ was rapidly implemented to assess the prevalence of eye disease, degree and burden of vision impairment, and resource requirements for specific ophthalmologic needs.

In this report, we describe the detailed personnel requirements, ophthalmic equipment and supply procurement strategy, screening eye clinic design and flow that could be utilized in the screening setting, and a modular design of the clinic for clinical evaluation and teaching of basic components of the eye exam to in-country staff. In addition, infection control precautions and personal protective equipment guided by our initial assessment of EBOV by RT-PCR in both intra- and extraocular fluid^8^ are described for the screening of survivors in the EVD outbreak setting. Given the recent and ongoing outbreaks in the Democratic Republic of Congo (DRC), there likely will be an ongoing need to understand the resources, equipment, expertise and systems needed for EVD survivor care beyond the critical importance of acute EVD care in resource limited settings.

## Methods

### ELWA Hospital setting, personnel requirements, and patient recruitment

Eternal Love Winning Africa Hospital is a health facility founded by Serving in Mission (SIM) in 1965 located in Paynesville City, Monrovia, Liberia. Faith-based organizations (Samaritan’s Purse and SIM) developed and operated the ELWA (ELWA-1 and 2) Ebola Treatment Units (ETUs) from June to August 2014 with the support of Medecins sans Frontieres-Belgium (MSF-B).[9] ELWA-1 and 2 cared for 69 patients from June to August 2014 and 53 (77%) were confirmed cases.[9] An EVD Survivor Clinic was initiated in January 2015 as a response to the emerging EVD systemic sequelae increasingly observed in survivors. EVD Survivor services provided include general adult and pediatric medicine, surgical consultation, rheumatology, psychosocial counseling, prenatal care, imaging, and laboratory studies.

In-country Liberian personnel requirements required at ELWA Hospital during development of the eye clinic included registration (2 individuals), interpreters/ medical assistants (4), and primary care physicians (2) involved in direct EVD patient care in an Ebola Treatment Unit (ETU) setting. Emory University and United States HCWs included three ophthalmologists and one infectious disease provider familiar with EVD survivor sequelae and EVD patient care in an ETU setting. In addition, engagement with the Ministry of Health and Sanitation Liberia was important for ongoing discussion of the patient findings, determination of resources needed for clinical ophthalmic care, and temporary Liberian medical licensure for physicians involved in EVD survivor ophthalmic care.

EVD survivors who were receiving ongoing care at ELWA Hospital Survivor Clinic were offered an ophthalmic evaluation during the screening program period at the time of their general medical care evaluation. In addition, EVD survivor associations were engaged by ELWA clinicians regarding the screening effort prior to the eye care program so that they could receive an evaluation, care, or referral as needed. The presence of an eye clinic for screening, treatment, and referral was also directly communicated with existing EVD survivor clinics in Monrovia (e.g. Médecins Sans Frontières) for patient evaluation.

### Ophthalmic equipment, medication procurement, and clinical form documentation

A supply list for the ophthalmic clinic was generated to include all clinical equipment required for the eye exam (Snellen visual acuity charts, Tumbling “E” chart, penlights, Tonopen for intraocular pressure assessment and disposable Tonopen tip covers, portable slit lamp, indirect ophthalmoscopes with condensing lens and appropriate voltage converters; Table 1). Because of the uncertain availability of topical medications required to treat uveitis and uveitis sequelae, as well as other treatable ocular diseases (i.e. ocular hypertension), supply lists were generated for topical medications required for clinical examination (i.e. topical proparacaine, 2.5% phenylephrine, 1% tropicamide), as well as for therapeutic use for ocular inflammatory disease (i.e. topical corticosteroid, topical cycloplegic agents). Multiple sources were researched for ophthalmic equipment and medications to limit the overall cost; these sources included industry donations, discount websites (eBay, Amazon) and international ophthalmic distributors where discounts could be utilized for the purchase of ophthalmic equipment. In addition, humanitarian pricing was solicited for capital equipment to minimize funding requirements for procurement. While donations were of benefit for the near-term goals of the project, longer term strategies for medication procurement including supply chain, cost, and source of revenue are considerations, as donations rely on supportive industry sponsorship, availability, and timing of donations from industry partnerships.

**Table 1:** Ophthalmic supply list and procurement strategies.

Item	Cost	Value (Approximate)	Method of Procurement
**Equipment**			
Portable slit lamp and 3 bulbs	$6600	$7100	Discounted from distributor
Indirect ophthalmoscope	$1300	$1300	eBay Discount website
Tonopen	$1200	$1200	eBay Discount website
Penlights	$50 (Qty: 7)	$50	Amazon
Slit lamp (Used)	Donated	$1500	Clinic donation
Retinoscope/ Muscle light	Donated	$600	Clinic donation
Condensing lenses	$400	$600	Discounted from distributor
iPhone 6	$400	$400	Retail
**Supplies**			
Visual acuity charts (Tumbling “E” and Snellen charts)	$30 (Qty: 9)	$100	Discounted from distributor
Near visual acuity cards	$15 (Qty: 2)	$15	Retail
Tonopen covers	$150 (Qty: 600)	$150	Amazon
Tonopen batteries	$30 (Qty: 6)	$30	Amazon
Alcohol swabs	$10 (Qty: 100)	$10	Retail
Cotton swabs	$20 (Qty: 100)	$20	Retail
Gloves (S, M, L)	$80 (Qty: 8 boxes)	$80	Retail
Eye shields	$10 (Qty: 20)	$10	Retail
Hand sanitizer	$40 (Qty: 2)	$40	Retail
Gowns	$35 (Qty: 2 boxes of 50)	$70	Amazon
**Medications**			
Topical corticosteroids	Donated (Qty: 300 bottles)	$30,000	Industry foundation donation
Glaucoma medications	Donate (Qty: 150 bottles)	$25,000	Industry foundation donation
Dilating drops/ cycloplegic agents	Donated (Qty: 50 bottles)	$25,000	Industry foundation donation
Oral acetazolamide (Diamox)	$200 (Qty: 100 tablets)	$200	Retail
Phenylephrine	$800 (Qty: 10 bottles)	$800	Retail
Atropine	$525 (Qty: 75 bottles)	$525	Retail
**TOTAL**	**COST: $11,895**	**~VALUE: 94,800**	

**Fig 1:**
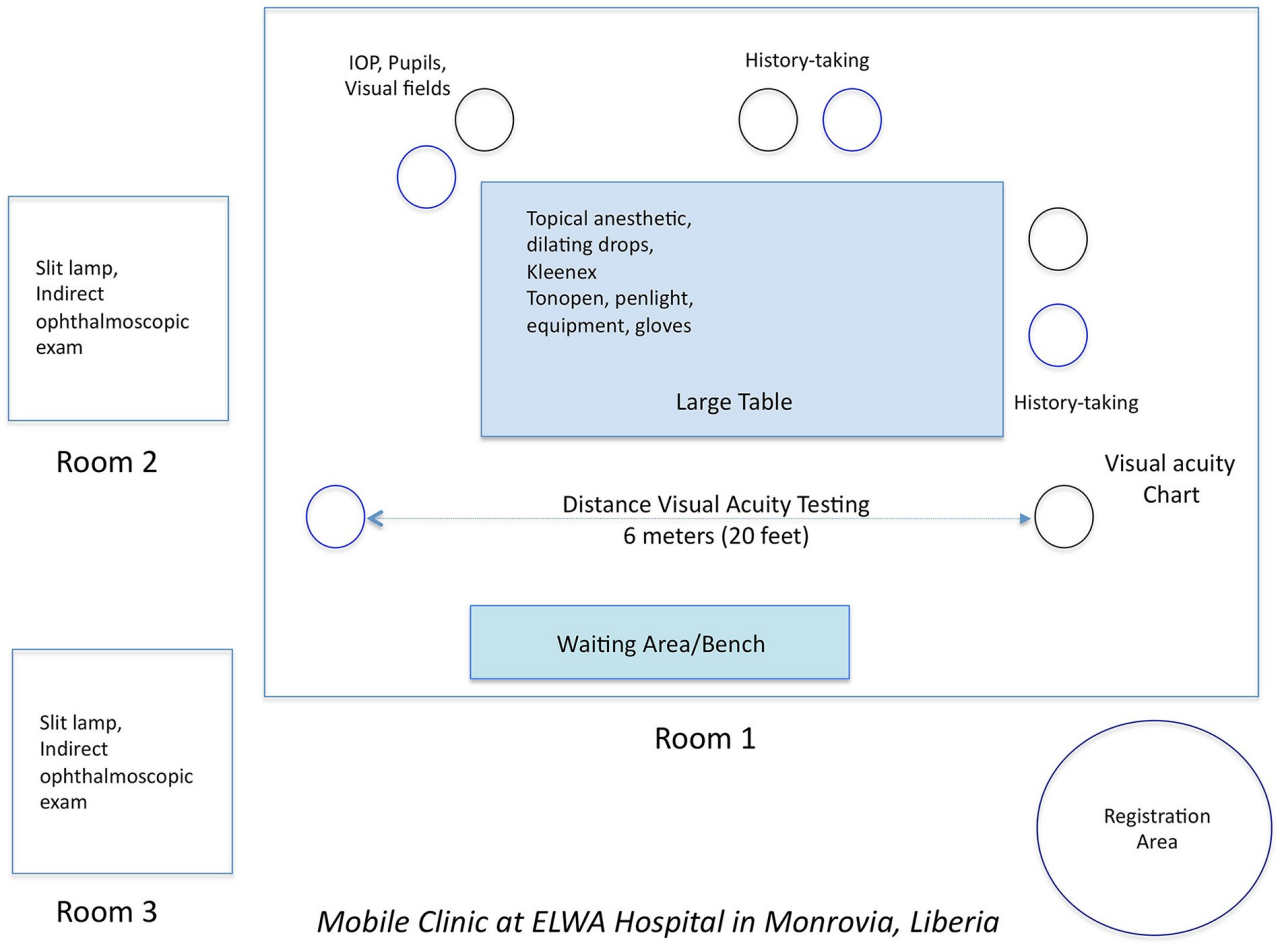
Blueprint of screening clinic flow in a large conference room at ELWA Hospital in Monrovia Liberia. A registration area is separate from the clinic area for triage, registration, and temperature monitoring. In Room 1, patients had their histories taken, visual acuity testing, intraocular pressure and visual field assessment prior to dilation. Slit lamp examination, indirect ophthalmoscopy, and counseling were individualized in Rooms 2 and 3.

**Fig 2:**
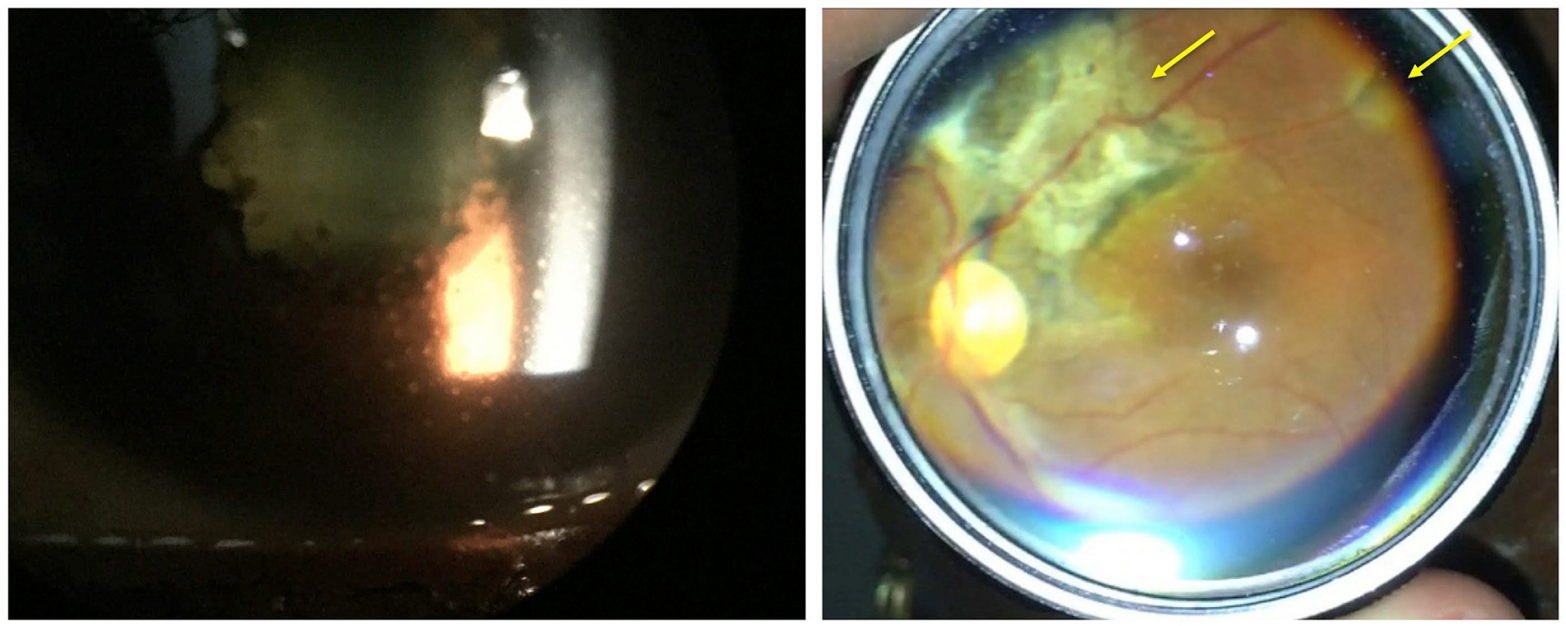
Slit lamp photograph of an EVD survivor with panuveitis shows keratic precipitates on the cornea with posterior synechiae (left). Treatment with oral prednisone, topical difluprednate and atropine were required. Another EVD survivor shows multifocal chorioretinal scarring (yellow arrows) indicative of posterior uveitis. All photographs are taken with an iPhone either at the slit lamp (left) or with a condensing lens (right).

Clinical forms were designed to capture systemic sequelae relevant to EVD survivors as well as a complete ophthalmic examinaztion including ocular vitals (visual acuity, intraocular pressure, extraocular motility, confrontational visual fields, and pupil exam), slit lamp examination, and dilated fundus exam (Fig 1). Standardized descriptors of uveitis and complications of uveitis according to Standardization of Uveitis Nomenclature. iPhone devices were utilized with the slit lamp eyepiece for anterior segment photography and iPhone devices with 28-diopter condensing lenses were utilized for fundus photography for optic nerve, macular lesions, and mid-peripheral retinal lesions (Fig 2).

### Screening eye clinic design, clinic flow, and modular design

A conference room with a large table and two separate patient examination rooms were utilized and partitioned into task-specific stations including 1) history-taking 2) pupillary evaluation, ocular motility, visual field testing, intraocular pressure, and dilation 3) slit lamp examination, indirect ophthalmoscopy, photography, and counseling including description of medication regimens, referral to other providers, and follow-up visit (Fig 1).

### Infection control and personal protective equipment

Patients checked in at a registration desk with HCWs wearing PPE that included a face shield, fluid impervious gown and gloves. EVD survivor certificates were verified at the registration desk. Forehead temperature with an infrared thermometer was measured. Patients with temperatures less than 38 degrees Celsius (100.4 degrees Farenheit) were evaluated. Ophthalmic examination was deferred in patients with a fever or any systemic signs of EVD (fevers, rigors, myalgias, diarrhea, vomiting) until complete medical evaluation could be performed. HCWs in the screening eye care facility wore fluid-impervious gowns and gloves. Strict handwashing precautions were performed with 0.05% chlorine and all equipment (portable slit lamp, Tonopen) was disinfected with alcohol between patients. At the time the clinic was conducted, limited information was available on the kinetics of EBOV in the tear film, specifically whether the tear film was fully clear of EBOV. EBOV persistence in tear film has been documented at 33 days post acute infection.[10] For this reason, a protective gown and gloves were utilized to avoid any potential skin exposure, as well as strict hand disinfection between patients.

### HCW evacuation plan, safety and regulatory considerations

During the West African EVD outbreak, there was heightened travel regulatory approval to ensure safety of the HCWs and the public in the rare event of EBOV infection. Emory University physicians underwent several tiers of university regulatory approval including Department Chair, Dean, Chief Executive Officer of The Emory Clinic, and the Emory University Executive Travel Safety Committee (Table 2). After returning from West Africa, HCWs underwent 21-day temperature and EVD symptom monitoring according to Georgia Department of Public Health, Centers for Disease Control and Prevention, and Emory University Hospital guidance. Travel medical and evacuation insurance in the event of acute illness was also purchased for United States-based HCWs.

**Table 2:** Partnerships, approval, and regulatory requirements.

In-country providers and staff from Liberia	Emory Personnel	Emory Approvals
Engagement with Ministry of Health and Sanitation, Liberia	Support of clinical and administrative leadership	Chair, Department of Ophthalmology
Registration Desk for temperature monitoring/ medical records (2)	Ophthalmologists (3)	Chief Medical Officer, The Emory Clinic
	Infectious Disease (1)	
Interpreters / Medical Assistants (4)	Occupational Health for daily temperature monitoring following return from West Africa	Dean, Emory University School of Medicine
Primary care physicians (2)		Executive Travel Safety Committee, Emory University (Multiple clinical and administrative leaders from Emory University), Office of Graduate Medical Education
**Local, State and Federal monitoring requirements following return from Ebola-affected area**	**Other Administrative and Travel Requirements**	
Emory University Hospital	Temporary Medical License from Liberia Ministry of Health	
Georgia Department of Public Health	Administrative Approval from Serving in Mission	
Centers for Disease Control and Prevention	Travel medical and evacuation insurance for health care providers	

## Results

With thorough supply lists designed for ophthalmic examination in a resource-limited setting, multiple sources were utilized for procurement and judicious resource use, as summarized in Table 1. Ten different sources were utilized to procure supplies and medications utilizing a combination of cost reduction from distributors, donations where possible, and retail when necessary or products were unavailable at a reduced cost. Industry donations included topical corticosteroids, ocular hypotensives and cycloplegic agents to treat acute uveitis. Two slit lamps were utilized for patient evaluation including a portable slit lamp and free standing slit lamp, which was deconstructed, transported following breakdown of its components into a suitcase and rebuilt for clinical use. iPhone photography through the slit lamp oculars and iPhone photography with a 28-diopter condensing lens were utilized for documentation of anterior segment and posterior segment pathology.

The estimated value of medications, equipment, and supplies for the screening eye clinic was $94,800 and all materials were procured for $11,895, a discount of approximately 87% of the total value of goods utilizing multiple methods of procurement, donations through the generosity of various entities (i.e. clinic, industry foundation), as well as humanitarian discount pricing when possible.

Components of the clinic design included modular stations for 1) history, review of systems, and general physical examination 2) visual acuity measurement 3) pupil, motility, visual fields, intraocular pressure measurement and dilation 4) slit lamp and indirect ophthalmoscopy, photography, and counseling. These clinical stations provided opportunities for both clinical care and instruction of the basic eye exam for in-country HCWs and were recorded on standardized case report forms for documentation and follow-up (Appendix). In particular, patients were evaluated for findings suggestive of uveitis. Specific complications of uveitis assessed included posterior synechiae, band keratopathy, and cataract. The use of indirect ophthalmoscopy and hands-on experience with in-country eye care provider allowed the evaluation of patients for retinal and optic nerve disease. Following the clinic setup and operations in April 2015, the ophthalmic clinic, equipment and medications remained available for ELWA hospital physicians and staff for patient follow-up examination and treatment. In addition, EVD survivors who were not specifically assessed during clinic operations could be evaluated thereafter.

Based on this setup, 96 EVD survivors were successfully examined, treated or referred when appropriate.[11] To ensure appropriate transfer of care and follow-up, patients who needed ongoing follow-up were discussed with physicians permanently stationed at ELWA Hospital. Of the EVD survivors screened, EVD-associated uveitis was identified in 21 survivors and EVD-associated optic neuropathy was observed in 4 patients. Patients with uveitis were found to have vision impairment in 60% of eyes, while severe vision impairment (i.e. World Health Organization criteria for severe vision impairment, 20/400 or poorer) in approximately 40% of individuals. Our screening clinic setup was also sufficient to classify EVD survivors who were screened into anterior, posterior, and panuveitis classificatons of uveitis. Visual acuity qualified as severe visual impairment by World Health Organization criteria in 38.5% of affected eyes.[11] Our findings are in agreement with recent literature on the burden and vision impact of eye disease in EVD survivors who developed uveitis as a sequelae of their acute EVD infection.[12,13]

Based on these initial experiences in Liberia and developing a screening eye clinic in partnership with ELWA Hospital, the portability, structure, and equipment of the ophthalmologic exam and consultation setup was discussed with the World Health Organization, Ministry of Health and Sanitation Sierra Leone National Eye Program and partnering non-governmental organizations including Partners in Health, and Médecins sans Frontières. Over 2,700 EVD survivors in Sierra Leone have been screened through the National Eye Program with clinical form development, treatment protocols and guidance on uveitis prevalence and disease burden.[7] Information attained from early experiences in Liberia developed in collaboration with ELWA Hospital were communicated with WHO and CDC officials involved in the EVD outbreak response as attention shifted from the acute outbreak to EVD survivor care.[2,14]

## Discussion

As a consequence of the West African EVD outbreak from 2014–2016, thousands of EVD survivors remain at-risk for systemic and ocular sequelae mandating equipment and resources for ongoing health care needs.[11–15] Given the identification of uveitis in over 20% of EVD survivors with resultant severe vision impairment or blindness in nearly 40% of individuals, the development of approaches to the high proportion of individuals at-risk to ophthalmic sequelae is needed.[11–13,15] In addition, Ebola virus persistence in immune privileged organs including the eye have presented ongoing public health considerations.[4, 16–20] In addition to EVD survivors within West Africa, more recent EVD outbreaks in DRC within 2018, including an ongoing EVD outbreak which has now eclipsed 700 infected individuals within eastern DRC, underscore the immediate need to understand the equipment, resources, and systems needed to rapidly evaluate EVD survivors with ophthalmic complaints from care and public health standpoints.

The urgent development of a screening eye clinic during the EVD outbreak setting was mandated because of initial experiences with United States HCWs who developed sight-threatening disease[4] and extremely concerning reports of eye disease from West Africa following recovery from acute EVD. In this context, the detailed requirements requiring collaboration between local ophthalmologists and eye care providers, technical experts in ophthalmic subspecialty care related to uveitis and retinal disease, supplies, medications, and rapid development of management protocols were needed to successfully garner information about a novel disease process and emerging problem. All of these personnel and resource requirements were necessary for ophthalmic screening in a resource-limited setting. Staffing included both Emory Eye Center ophthalmologists and infectious disease HCWs and in-country partners included administration, clinical leadership, and staff of SIM / ELWA Hospital.

Meetings with local eye care providers in West Africa have been subsequently continued to discuss the vision-threatening complications that are currently being studied by investigators in Sierra Leone, Liberia, and Guinea. Within Sierra Leone, the Ebola Virus Persistence in Ocular Tissues and Fluids (EVICT) Study is ongoing to evaluate the prevalence of Ebola virus in ocular fluids of EVD survivors anticipating cataract surgery. Recent results have describing the safety and feasibility of vision-restorative cataract surgery in EVD survivors with a sequential assessment of aqueous humor fluid for EBOV by RT-PCR and subsequent cataract surgery. The National Institutes of Health PREVAIL Study and Eye Sub-study is a prospective, controlled, natural history evaluation of EVD survivors and asymptomatic close contacts with patients receiving ongoing care with local Ministry of Health eye care providers and surgeons.[21] The PostEbogui Study in Guinea has also characterized EVD survivor sequelae including ocular disease.[13]

Besides the development of a screening clinic to carry out detailed screening with standardized reporting of uveitis complications in the cohort of EVD survivors, our setup also included consideration of the environment and safety concepts including PPE tailored to the potential risk of EBOV exposure health care providers involved in the clinic. Our knowledge at the time of the screening clinic design included the following: 1) Ebola virus may be harbored in ocular fluid at least 100 days post acute EVD diagnosis [4]; 2) our assessment of a HCW with panuveitis associated with Ebola virus in ocular fluid demonstrated that the conjunctiva/ tear film tested negative for EBOV RT-PCR at the time the ocular fluid was EBOV-positive by RT-PCR [4] and; 3) Conjunctiva may show delayed clearance of EBOV from the ocular surface with a conjunctival swab positive for EBOV RNA by RT-PCR at 33 days post acute EVD diagnosis.[10] This knowledge led to our use of modified PPE to protect HCWs while balancing other practical issues including dehydration and potential heat exhaustion with heavy PPE required for ETUs with active EVD patients. Additionally, patients were screend with systems and temperature check to ensure patients were not actively ill. More recently, the EVICT study, which involves sampling of ocular fluid with the potential for viable EBOV, has required full PPE given the potential for EBOV persistence in intraocular specimens.[8]

The development of screening clinics in resource-limited settings has been reported in India, sub-Saharan Africa and rural parts of Asia.[22, 23] Predominantly designed to evaluate blinding cataract, these clinics have not necessarily required equipment for dilated funduscopic examination. The clinic in Liberia addresses more equipment intensive needs to evaluate eyes for uveitis and retinal disease including indirect ophthalmoscopy. Within Liberia and Sierra Leone prior to the recent West African EVD outbreak, there were few indirect ophthalmoscopes available for peripheral retinal examination (i.e. less than five functional indirect ophthalmoscopes within Liberia and Sierra Leone). To properly evaluate for posterior uveitis and retinal scarring recently observed in EVD, indirect ophthalmoscopes were procured for care and for training.

The goals of the screening clinic that were accomplished included identification of EVD survivors requiring immediate eye care, referral to eye care providers, and informing implementing partners and public health organizations regarding technical needs of equipment for ophthalmic care needs. Patients with eye disease were referred to MOHS providers for ongoing care and lessons learned were utilized to inform EVD screening in Sierra Leone given the urgency of uveitis care with the potential for severe vision loss if left untreated. Moreover, our findings in addition to recent findings by other organizations, have been informative for educational symposia conducted in Sierra Leone related to eye disease in Ebola survivors and the design of management protocols for distribution to health care workers in West Africa. In addition, our report of the ophthalmic complications with a 22% rate of uveitis and impact on vision^10^ through the implementation of this screening clinic, has been consistent with other studies estimating a prevalence of uveitis between 18% and 34% of EVD survivors.[12,13,15,21]

Importantly, no symptoms of EVD developed in the HCWs who participated in ophthalmic assessments of EVD survivors and underwent 21-day symptom and temperature monitoring following re-entry in the United States. While the exposure of HCWs to EVD survivors with uveitis was short-term in this evaluation of EVD survivors in Monrovia, Liberia, there remain no known risks of EBOV transmission through casual contact including the ophthalmic examination of EVD survivors, underscoring the importance of safety of ophthalmic examination with appropriate PPE. However, the potential risk of EBOV persistence in ocular fluids immediately following acute EVD and during early disease convalescence less than 3 month remains unknown. Despite these uncertainties, efforts are ongoing to better understand and reduce stigma associated with EVD, which is an ongoing concern amongst EVD survivors.[24] Further information from the EVICT study[8], which is characterizing EBOV persistence in extra- and intraocular fluids of EVD survivors anticipating eye surgery, and the National Eye Institute-funded PREVAIL Eye Sub-Study will inform guidance related to eye examinations and diagnostic and therapeutic invasive ophthalmic procedures.

### Conclusion

In summary, through a collaboration with United States-based ophthalmologists and leadership of ELWA Hospital, in conjunction with eye care specialists from the Ministry of Health, Liberia we were able to develop and rapidly implement a screening eye clinic for EVD survivors. The procurement of equipment through a variety of sources, modular clinic design, and attention to PPE appropriate for the ophthalmic examination setting were necessary to provide efficient care, referral, and safety for patients and health care providers. Lessons learned from this clinic were communicated to other countries with thousands of EVD survivors and underscore the importance of ongoing understanding of eye disease in EVD survivors, particularly given the ongoing outbreak in eastern DRC.
